# Genetic Cluster Analysis for HIV Prevention

**DOI:** 10.1007/s11904-018-0384-1

**Published:** 2018-02-19

**Authors:** Mary Kate Grabowski, Joshua T. Herbeck, Art F. Y. Poon

**Affiliations:** 1 Department of Epidemiology, Johns Hopkins Bloomberg School of Public Health, Rakai Health Sciences Program, Baltimore, MD USA; 2 International Clinical Research Center, Department of Global Health, University of Washington, Seattle, WA USA; 3 Department of Pathology and Laboratory Medicine, Western University, London, ON Canada

**Keywords:** Prevention, HIV, Cluster identification, Genetic similarity clusters, HIV epidemiology

## Abstract

**Purpose of Review:**

This review summarizes the use of genetic similarity clusters to understand HIV transmission and inform prevention efforts.

**Recent Findings:**

Recent emphases include the development of real-time cluster identification in order to interrupt transmission chains, the use of clusters to estimate rates of transmission along the HIV care cascade, and the extension of cluster analyses to understand transmission in the generalized epidemics of sub-Saharan Africa. Importantly, this recent empirical work has been accompanied by theoretical work that elucidates the processes that underlie HIV genetic similarity clusters; multiple studies suggest that clusters are not necessarily enriched with individuals with high transmission rates, but rather can reflect variation in sampling times within a population, with individuals sampled early in infection more likely to cluster.

**Summary:**

Analyses of genetic similarity clusters have great promise to inform HIV epidemiology and prevention. Future emphases should include the collection of additional sequence data from underrepresented populations, such as those in sub-Saharan Africa, and further development and evaluation of clustering methods.

## Introduction

Within epidemics, certain subgroups may contribute disproportionately to epidemic growth, and specific clinical, demographic, or behavioral factors can define these subgroups [1–3]. Historically, risk factors linked to high relative rates of HIV transmission have been identified with survey-based methods [4, 5], or with partner-based studies that provide per-act estimates of individual-level transmission or acquisition risk [6, 7]. Recently, analyses of HIV gene sequences have gained popularity as a means to identify transmission risk factors. Such molecular epidemiological studies of HIV are now common in the concentrated epidemics of Europe and North America, due primarily to the post hoc use of *pol* genotypes (gene sequences) available from routine screening for resistance to antiretroviral therapy [8]. In the most common type of approach, genetic clusters (sets of closely related sequences) are identified, and subsequent analyses are based on these clusters. For instance, phylogenetic studies of clusters in North America and Europe have revealed (and confirmed) the importance of the men-who-have-sex-with-men (MSM) subgroup [9, 10] and of early HIV infection [11–13], in driving HIV transmission.

HIV phylogenetics and molecular epidemiology have experienced substantial recent advances, including in our understanding of genetic clusters and how their study can inform HIV prevention. In this commentary, we review underlying concepts of clustering relevant to HIV molecular epidemiology, report on examples of application of clustering methods to questions of HIV prevention, including the development of a real-time pipeline for HIV phylogenetic analyses, and discuss recent findings from HIV phylogenetic studies in sub-Saharan Africa. We note this is not intended to be a comprehensive review of the field. Furthermore, we discuss some open issues related to the use of genetic clusters, including the following questions: What is cluster growth, and how do we determine whether clusters are growing? Are genetic clusters enriched with certain individual traits or risk factors relative to non-clustered sequences? Can these traits be assumed to represent increased rates of transmission?

## What Is a Cluster?

In general, a cluster is a group of objects that are closer (more similar) to each other than to objects outside of the group. Selecting a cutoff that determines whether an object belongs to the cluster is a subjective decision that is the responsibility of the investigator. In the context of infectious disease epidemiology, cases can be clustered in space (e.g., a hospital ward), clustered in time (e.g., an excessive number of diagnoses in a short time interval), or in both space and time. An implicit assumption is that the majority of infections do not belong to clusters, which corresponds to an agglomerative clustering approach where every observation initially belongs to a cluster of one. Clusters can also be defined by the genetic similarity of infections (genetic clusters). This approach can be very useful when there is an abundance of pathogen gene sequences routinely collected for the clinical management of infection, when spatial information is incomplete, and when times of diagnosis are less informative because of a long asymptomatic period of infection. HIV-1, tuberculosis, and hepatitis C virus epidemics are canonical examples of infectious diseases that exhibit these characteristics.

**Fig. 1:**
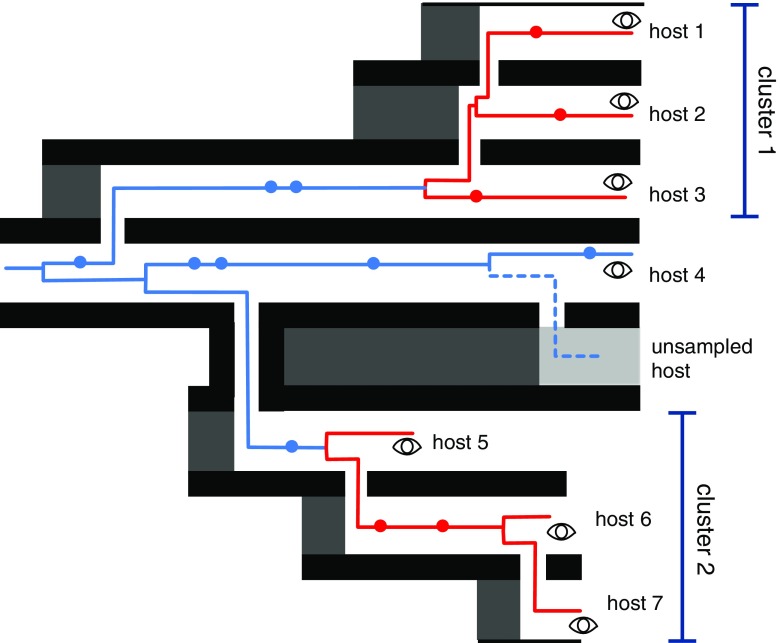
Schematic diagram of HIV-1 transmission and evolution. Each horizontal “track” represents a host individual separated by thick lines representing barriers that are crossed by virus transmission. The red lines represent virus lineages, and dots represent genetic differences that accumulate along each lineage. Events unfold over time from left to right. The sampling (observation) of lineages by sequencing is represented by the open eye symbols. Lineages that may be included in clusters are highlighted in red. Hosts 1–3 are related by a recent rapid series of transmissions and form cluster 1 because of limited genetic divergence since transmission. Host 4 appears to be distantly related to any other infection, although it would be similar to an unsampled lineage. Hosts 5–7 are sampled soon after becoming infected and thus form a second cluster despite a substantial time between the transmission events

Defining a genetic cluster, for the purposes of molecular epidemiology described here, requires a measure of similarity or distance between two sequences covering the same region of the genome (homologous sequences). For instance, there are many genetic distance measures that take sequences as inputs [14] (Fig. 1). The more complex measures accommodate variation in the rates for different types of nucleotide substitutions. One limitation of these distance measures is that they can only utilize the information contained in two genetic sequences to adjust these rate parameters. Clusters can be assembled from all pairs of sequences within a threshold distance of each other—this is the approach taken by the software HIV-TRACE [15]. The pairwise distances for a set of sequences can also be converted into a tree (phylogeny) that approximates how the sequences are related through common ancestors; the neighbor-joining algorithm is a popular technique for this conversion. This phylogeny makes it possible to define clusters of infections that are assumed to be descendents from a common source [16]. In addition, the phylogeny defines another distance measure in the form of the total branch length on the path from one sequence to another, i.e., the patristic distance. A common practice in HIV phylogenetics is to remove amino acid sites that are associated with antiretroviral resistance mutations *prior* to reconstruction of phylogenetic trees and identification of clusters. This is done to minimize the possibility or error introduced by convergent evolution (i.e., the independent evolution of similar traits due to selective pressure).

Phylogenies can also be reconstructed by maximum likelihood (ML) for a given model of evolution [17]. Since this approach jointly evaluates all the genetic sequences instead of two sequences at a time, it is more capable of accounting for variation in rates of evolution. On the other hand, ML methods are much slower to compute and it is not known whether the improved accuracy translates to more informative clusters [18]. Accurately reconstructing a viral phylogeny is a difficult problem because we must discriminate among an enormous number of possible trees relating a given set of infections [19]. A standard method for evaluating the uncertainty in phylogeny reconstruction is to resample the gene sequences at random with replacement (nonparametric bootstrap sampling) to generate false replicates [20]. If the same ancestor-descendant relationship (as defined by the branching pattern of the phylogeny) appears in most of the trees inferred from these replicates, then we have a high level of confidence that those infections (pathogen lineages) are more closely related than other infections in the sample population, given the data. This quantity is known as the bootstrap support value and is frequently used as a criterion for clustering sequences. Because this support value does not express how similar the infections are, it is frequently used in combination with a genetic distance [21, 22]. The computational burden of reconstructing many ML trees may preclude nonparametric bootstrap sampling; however, several fast methods have been developed that can produce good approximations of bootstrap values [23]. Aside from these differences in accuracy and computational burden, the same methods for defining clusters apply equally to ML and distance-based phylogenies. Lastly, some studies employ Bayesian methods to generate a random sample of phylogenies in proportion to their posterior probability [24], which is proportional to the product of the likelihood and the investigator’s prior belief. The Bayesian posterior probability associated with internal branches can serve the same purpose as bootstrap values for clustering [25].

## Examples of the Use of Clusters in HIV Prevention

### Real-time Cluster Identification

A current interest in HIV molecular epidemiology is the application of sequence-based analyses for real-time prevention efforts, in order to interrupt ongoing transmission chains. One promising approach includes estimates of priority for directed intervention that are based on networks of genetic similarity [26]. Another approach, from British Columbia (BC), combines the diagnosis, genotyping, and genetic similarity analyses into an automated pipeline; we describe this approach in greater detail here. All clinical HIV resistance genotyping in BC, and the majority of resistance tests in Canada, is performed by the laboratory program at the BC Centre for Excellence in HIV/AIDS (CFE). For all persons in BC, HIV resistance genotypes and clinical variables, such as viral loads and drug regimens, are integrated with demographic and risk factor information into a population treatment database. This comprehensive centralization within a “single-payer” health care system afforded a unique opportunity to implement an automated monitoring system that uses a phylogenetic clustering method to identify potential transmission outbreaks in near real-time [27]. In December 2012, epidemiologists and public health officers from the BC Centre for Disease Control met with members of the CFE laboratory program to discuss the development of a near real-time monitoring system based on this treatment database. A cached database query (termed a virtual table or “view” for an Oracle database) was implemented by database programmers at the CFE to integrate anonymized data, including HIV genotypes and sample collection dates, from multiple tables in the database in a tightly controlled and reproducible manner. Access to this view was password-protected and restricted to a small number of Oracle user accounts and IP addresses on the local CFE network, which is behind a dual firewall that blocks all remote network requests.

New genotype records were detected by querying the Oracle view for any records with a collection date that was more recent than the date and time of the last transaction. A positive result triggered the system to download the entire contents of the view from the database to a secure workstation running the monitoring system. The challenge was to develop a pipeline for the phylogenetic analysis of this massive dataset (over 30,000 sequences) that was sufficiently rapid for “real-time” monitoring. Two simple methods were employed to minimize computing time. First, the system used pairwise alignment of sequences against the HIV-1 *pol* reference sequence (HXB2) and excluding any insertions relative to this reference. This is the same approximation method used by HIV-TRACE. Second, the system used the FastTree 2 program for a fast approximate reconstruction of phylogenies by maximum likelihood [29]. Even though this program is orders of magnitude faster than more accurate programs based on ML, it still required over an hour to build each tree relating the entire sequence database. Although this approach should provide more accurate estimates of the evolutionary distance between sequences, a recent simulation study indicates that the pairwise genetic distance method employed by HIV-TRACE is nearly as effective while running in seconds [18].

By mid-2013, a functional real-time system had been implemented and was automatically generating reports to the laboratory director whenever new sequences were deposited in the database (almost daily). On October 2013, the public health and laboratory personnel began a series of monthly teleconference meetings to discuss outputs from the monitoring system. Monthly reports were requested by public health, and the content and format of these reports was gradually developed over a period of several months. One of the major challenges in developing this report template was the use of network diagrams for genetic clusters, in which nodes representing individuals are connected by “edges” to indicate that their HIV infections are genetically similar. These network diagrams resemble epidemiological contact networks where edges between individuals represent past interactions with a high risk of transmitting disease. Although genetic similarity may be correlated with the contact structure of the population, we cannot expect the respective networks to have the same shape. Annotating network diagrams by the most recent plasma viral load (node size) and presence of drug resistance mutations (node color) were well received by the public health officers, as this visualization scheme highlighted clusters with greatest predicted risk of onward transmission.

The role of this real-time monitoring system in detecting an outbreak of transmitted HIV drug resistance in Vancouver was recently reported as an implementation case study [27]. In brief, a cluster of 25 individuals (predominantly men who have sex with men) had grown by 11 new cases over a period of three months. Eight of the 11 new cases carried the same HIV mutation conferring resistance to first generation non-nucleotide reverse transcriptase inhibitors. A provisional report was issued to the provincial health authority, resulting in a formal outbreak investigation and enhanced public health follow-up in the affected subpopulation over the subsequent two months. Over the following year, the cluster continued to accumulate new cases but the frequency of transmitted drug resistance was significantly reduced.

### Transmission Along the Cascade of Care

One consistently proposed use of genetic cluster analysis has been to guide HIV prevention strategy—to increase the efficiency and effectiveness of interventions. Targeted prevention based on transmission risk factors was successfully demonstrated by Avahan, the India AIDS Initiative, which focused prevention on high risk groups in India and led to > 100,000 estimated HIV infections prevented in the general population between 2003 and 2008 [30, 31]. Avahan identified epidemic drivers without phylogenetic analyses, and it may be that existing epidemiologic data adequately identifies groups at high risk for transmitting HIV in the USA as well. However, they may not. An example relates to the problem of identifying the stage of individual infections that are the source of the majority of new infections. In another study that was not based on phylogenetic analysis, the United States Centers for Disease Control estimated that most new HIV infections are transmitted from persons who are diagnosed and out of care [32] and that ~ 30% of new infections are from undiagnosed individuals. Yet phylogenetics can be used to address this specific question, or, at minimum, to augment the standard approach to understanding transmissions along the cascade of care. Recent such studies have focused on the resurgent HIV epidemic among men-who-have-sex-with-men (MSM) in the Netherlands [33, 34]. Notably, [34] focused on transmissions to MSM with confirmed recent HIV infection at time of diagnosis. The goals of this study were (1) to reconstruct the probable transmission events to these recipient MSM; (2) to estimate the proportion of these transmissions from different stages in the infection and care continuum; and (3) to estimate, via mathematical modeling, the proportion of infections that could have been averted through counterfactual prevention programs. Phylogenetic analysis of 1045 (anonymized) potential recipients included > 6000 total sequences from the Netherlands and an additional 700 contextual sequences obtained from the Los Alamos National Laboratory HIV Sequence Database. The resulting phylogeny was used to exclude potential pairs, leaving 903 phylogenetically probable transmitters to 617 recipients. Phylogenetic transmission probabilities were ascribed to each remaining pair based on independent data on “known” HIV transmission pairs that were previously confirmed through detailed interviews and subsequent phylogenetic analysis [35]. Linked clinical records, available over time, were used to identify the stage of infection of each probable transmitter during infection windows of recipients. From this, the probability of transmission for each stage in the HIV infection and care continuum was estimated at the population level. The role of undiagnosed infections in driving the Dutch epidemic was estimated: they found that 71% of probable transmissions were from undiagnosed MSM, 43% from those in their first year of infection, 6% from men who had initiated ART, and 1% from men with no contact to care for at least 18 months.

First, these results suggest that viral suppression due to ART is effective in decreasing transmission rates, as expected [36]. Second, 71% (from undiagnosed individuals) is substantially greater than the ~ 30% estimated by Skarbinski et al. for the USA (in 2009) (a comparison of the Dutch MSM population with the overall epidemic population of the USA, however). Third, they estimated that very few transmissions (1%) were attributable to temporary or permanent loss to follow-up, compared to studies from the USA that estimate > 50% of all transmissions among MSM originate from men not retained in care [32, 37]. However, the 40% of transmissions estimated to come from early infection was similar to that estimated by a phylodynamic analysis of MSM in Detroit (45%) [11].

Better understanding of the characteristics of persons who transmit HIV, including where they are located within the HIV infection and care continuum, and the factors that may impede their early diagnoses and effective treatment could allow public health officials to better target resources to maximize prevention impact.

### Application of Phylogenetics to Generalized HIV Epidemics of Sub-Saharan Africa

Despite accounting for more than two thirds of HIV infections and AIDS-related deaths worldwide, the African HIV epidemic is poorly represented within the phylogenetic literature [8]. Historically, most African HIV phylogenetic studies have focused on characterizing viral diversity and drug resistance from sparsely sampled populations in HIV treatment and care programs. Few studies have assessed HIV emergence and geographic patterns of viral spread and an even smaller number have assessed modern-day epidemic dynamics and risk factors for viral transmission.

Phylogenetic studies of African HIV epidemics reveal strikingly different clustering patterns compared to those observed in concentrated epidemics. Unlike American and European HIV phylogenies from specific outbreaks, injection drug users, or MSM populations, African HIV phylogenies are typically characterized by small cluster sizes (< 5 persons/cluster) with limited geographic and epidemiological substructure [8, 15]. For example, a multi-pronged analysis, including spatial, partner network, and phylogenetic analysis, [38] showed that within rural Ugandan villages, there were numerous small phylogenetic clusters, nearly half of which were two individuals residing in the same household, and that many of these small clusters were connected to other infections in different villages. The phylogenetic results suggested that HIV is frequently introduced in villages with limited onward transmission. These findings were supported by spatial analyses showing limited geographic clustering of viruses outside of household and in analyses of partner networks revealing a high probability of infection among those with sexual contacts outside their community, particularly among women. In another phylogenetic study of the Kenyan HIV epidemic, [39] found extensive geographic mixing between MSM living in coastal Kenya and the capital city, Nairobi.

The small cluster sizes observed in African epidemics are most likely the consequence of a low *sampling fraction*, defined here as the fraction of extant HIV infections in the studied population that are represented by HIV sequence data (see Box 1). The lower the sampling fraction, the more challenging it is to identify putative transmission chains, a requirement for source attribution studies aiming to identify high risk transmitters. Simulation studies also show that local network structure and parameters (e.g., the momentary degree distribution, assortativity) are difficult to discern at low sampling fractions [40]. This is a critical problem in African settings with large epidemics and limited surveillance, because the sampling fraction is usually under 10%. Compare this to the ATHENA cohort, which includes sequence data from 60% of all HIV-infected persons in the Netherlands [41]. There are ongoing efforts to improve sampling of African HIV sequences, including the PANGEA-HIV consortium [42] that has generated more than 16,000 partial and full-length HIV genomes from several well-characterized East and Southern African epidemics. Analyses of these data are presently ongoing.

**Table:** Box 1 Definitions of common terms in HIV genetic clustering

*Bootstrap support value*: An estimate of confidence in the accuracy of reconstructing a particular section of a phylogenetic tree. A section (subtree) is defined by a branching point in the tree that represents the common ancestor for certain sequences. *Cluster growth*: A genetic cluster “grows” when a reanalysis of a database results in the addition of sequences to a previously observed cluster, representing infections that have been sampled since the previous clustering analysis. To date, there is no consensus on how to quantify growth rates for comparison among genetic clusters. *Genetic cluster*: A group of gene sequences representing sampled individuals that are more similar to each other than to sequences from outside of the cluster. The composition of a cluster can be determined by an algorithm (set of rules), but these rules ultimately depend on one or more subjective criteria. *Genetic distance*: Any method for converting two genetic sequences into a number that measures how different they are. A simple example of a genetic distance is to count the number of differences between a pair of aligned sequences. *Sampling fraction*: Generally, this term refers to the proportion of individuals who have been sampled from the infected population. However, this definition is ambiguous because the number of sampled infections accumulates over time and may also refer to the proportion of diagnosed infections for which genetic sequences are available. In some models, the sampling fraction is the proportion of “extinct” virus lineages that were terminated due to being sampled with the assumption that the individual then receives effective treatment [28]. *Transmission network*: A graphic representation of the transmission history in a population of infected individuals. Each individual is represented by a node in the network. A directed edge (arrow) drawn between nodes indicates that the pathogen was transmitted. A transmission network usually does not contain information about the timing of transmission events. Hence, it can be viewed as a “flattened” transmission tree. It is implicitly assumed that a transmission network represents a complete sample of infections in the population from the start of the epidemic to the present time. Genetic clusters are often equated with transmission networks; although they may be similar in shape, there are several reasons for discordance including incomplete sampling of infected individuals, variation in sampling times, and variation in rates of virus evolution	

The importance of sampling fraction is illustrated in two recent studies. In a phylogenetic assessment of age-disparate partnership and HIV risk in South Africa [43], the sampling fraction of the local HIV-infected population was no more than 4%. Nevertheless, the authors identified putative heterosexual transmission pairs from phylogenetic clusters, assuming that within clusters, older partners always transmitted to younger ones. Predictably, the authors concluded that older men were an important source of HIV infection to younger women, but because they did not account for low sampling and because of assumed directionality of transmission within clusters, the results provide limited insight into sources of HIV infection among young women. In another source attribution study, assessing the contribution of HIV transmission among MSM to HIV incidence in Nigeria, [44] explicitly account for incomplete and differential sampling within a complex coalescent population genetic framework. Here, the authors estimated that ~ 9% of new HIV cases among reproductive age women could be attributed to transmission from untreated infections among MSM.

Recombination is another critical problem, related but distinct to sampling fraction, in phylogenetic studies of African epidemics. In some settings, inter-subtype recombinant infections range from ~ 20 to 50% of all HIV infections [38, 39, 45]. Intra-subtype recombination is likely just as common but difficult to identify using existing statistical tools. Recombinant infections are problematic because they violate the assumptions of strictly bifurcating and non-reticulating lineage evolution that underlie most phylogenetic approaches; consequently, sequences that are identified as likely recombinants are almost always discarded from analytic datasets.

## Caveats About HIV Clusters

The emphasis within HIV molecular epidemiology on clusters (as defined by genetic similarity) has been influenced by qualitative observations and by simplifying assumptions. Observations (i.e., looking at pathogen phylogenies) have resulted in the assumption that an outbreak (whether spatial, temporal, or both) will commonly be reflected as a cluster. But the converse is not always true; a cluster does not always reflect an outbreak.

A related, and perhaps more implicit, assumption relates to the use of clusters to identify risk factors linked to high relative rates of transmission. One may postulate that all viral lineages in a phylogeny are cases of HIV infection (acquisition), but that only lineages in a cluster are likely cases of infection followed by transmission (on average, given sampling and linkage probabilities). Thus, the individual risk factors over-represented in clusters have been assumed to contribute more, on average, to transmission in an epidemic.

However, several recent studies have attempted to explicitly identify the processes through which genetic similarity clusters arise. For example, [46] demonstrated that the excess genetic clustering of individuals sampled in early infection is at least partially explained by the minimal genetic divergence experienced by such viral lineages (i.e., early infections are connected by short phylogenetic branch lengths to the transmitting individuals), rather than the expected elevated rates of transmission during acute HIV infection. Similarly, using epidemic simulations, [18] found that the majority of cluster identification methods were biased to detect variations in sampling rates among subpopulations, rather than variations in transmission rates. This study stressed that these variations in sampling rates may be due to the method of sampling in resource rich settings: high-risk individuals, who are already engaged in primary care, are more likely to be sampled than subpopulations with less access to primary care who may also be burdened by high rates of transmission. This suggests that the contribution of phylogenetic clustering studies to inform public health interventions and prevent transmission may be limited in such settings—without appropriate corrections for sampling in the statistical analysis of risk factors. The epidemic model used by [18] represents an epidemic scenario typical of concentrated epidemic dominated by MSM; sampling is perhaps not random with respect to risk or behavior and is driven by individuals themselves. It is unclear if similar patterns of clustering would be found in the generalized HIV epidemics of sub-Saharan Africa. In such epidemics, clustering studies are (perhaps) more likely to be carried out on community/population cohorts in which individuals are sampled irrespective of their individual risk. Examples of such community cohorts include the Rakai Community Cohort Study in Uganda, and those administered by the Africa Health Research Institute, the Centre for the AIDS Programme of Research in South Africa, the University of Washington Partners in Prevention/PrEP Study, and the Botswana Harvard AIDS Institute Partnership. Phylogenetic analyses (based on full HIV genomes) of these cohorts are underway by the PANGEA-HIV consortium [42]; the full genomes are expected to result in improved phylogenetic resolution [47, 48], although the question of what process is the primary driver of genetic clustering will remain.

An additional issue is when pairs of individuals within genetic clusters are interpreted as transmission partners absent additional epidemiological information. This tendency to equate genetic similarity with transmission at the level of individuals is particularly dangerous in settings where HIV is criminalized, since the same methods used to generate clusters have also been used in the prosecution of individuals for HIV transmission without disclosure of their infection status. The identification of group-level associations with phylogenetic clustering may also result in marginalization of vulnerable subpopulations, such as migrants. Hence, applications of genetic clustering studies to HIV-infected populations carry significant ethical and legal implications that have not been adequately addressed in the research community.

## Conclusion

Recent years have seen exciting advances in HIV molecular epidemiology, including the push for real-time analyses linked directly to public health interventions, and the application of phylogenetic approaches to describing patterns of transmission in the generalized epidemics of sub-Saharan Africa. Critically, the field has also begun to reevaluate key assumptions about clusters and the inferences that can be drawn from them. Evidence now supports greater caution is needed for the design and implementation of clustering studies that attempt to answer epidemiological questions. In short, analyses of viral gene sequences have great promise to contribute to our understanding of HIV transmission and to inform the targeted deployment of public health resources, yet the full potential of these methods will only be realized through continued development and critical evaluation.
